# Longitudinal Quantitative MRI Evaluation of Muscle Involvement in Amyotrophic Lateral Sclerosis

**DOI:** 10.3389/fneur.2021.749736

**Published:** 2021-11-24

**Authors:** Matteo Paoletti, Luca Diamanti, Shaun I. Muzic, Elena Ballante, Francesca Solazzo, Lia Foppoli, Xeni Deligianni, Francesco Santini, Silvia Figini, Niels Bergsland, Anna Pichiecchio

**Affiliations:** ^1^Neuroradiology Department, Advanced Imaging and Radiomics Center, Istituto di Ricovero e Cura di Carattere Scientifico (IRCCS) Mondino Foundation, Pavia, Italy; ^2^Neuro-Oncology Unit, Istituto di Ricovero e Cura di Carattere Scientifico (IRCCS) Mondino Foundation, Pavia, Italy; ^3^Department of Brain and Behavioral Sciences, University of Pavia, Pavia, Italy; ^4^Department of Radiology, Fondazione Istituto di Ricovero e Cura di Carattere Scientifico (IRCCS) Policlinico San Matteo, Medical School University of Pavia, Pavia, Italy; ^5^Department of Mathematics, University of Pavia, Pavia, Italy; ^6^BioData Science Center, Istituto di Ricovero e Cura di Carattere Scientifico (IRCCS) Mondino Foundation, Pavia, Italy; ^7^Radiology/Division of Radiological Physics, University Hospital of Basel, Basel, Switzerland; ^8^Biomedical Engineering, University of Basel, Allschwil, Switzerland; ^9^Department of Political and Social Sciences, University of Pavia, Pavia, Italy; ^10^Department of Neurology, Buffalo Neuroimaging Analysis Center, Jacobs School of Medicine and Biomedical Sciences, Buffalo, NY, United States; ^11^IRCCS Fondazione Don Carlo Gnocchi Organizzazione non lucrativa di utilità sociale (ONLUS), Milan, Italy

**Keywords:** ALS, muscle MRI, qMRI, fat fraction, edema, wT2, T2 mapping

## Abstract

**Background:** Biomarkers of disease progression and outcome measures are still lacking for patients with amyotrophic lateral sclerosis (ALS). Muscle MRI can be a promising candidate to track longitudinal changes and to predict response to the therapy in clinical trials.

**Objective:** Our aim is to apply quantitative muscle MRI in the evaluation of disease progression, focusing on thigh and leg muscles of patients with ALS, and to explore the correlation between radiological and clinical scores.

**Methods:** We enrolled newly diagnosed patients with ALS, longitudinally scored using the ALS Functional Rating Scale-Revised (ALSFRS-R), who underwent a 3T muscle MRI protocol including a 6-point Dixon gradient-echo sequence and multi-echo turbo spin echo (TSE) T2-weighted sequence for quantification of fat fraction (FF), cross-sectional area (CSA), and water T2 (wT2). A total of 12 muscles of the thigh and six muscles of the leg were assessed by the manual drawing of 18 regions of interest (ROIs), for each side. A group of 11 age-matched healthy controls (HCs) was enrolled for comparison.

**Results:** 15 patients (M/F 8/7; mean age 62.2 years old, range 29–79) diagnosed with possible (*n* = 2), probable (*n* = 12), or definite (*n* = 1) ALS were enrolled. Eleven patients presented spinal onset, whereas four of them had initial bulbar involvement. All patients performed MRI at T0, nine of them at T1, and seven of them at T2. At baseline, wT2 was significantly elevated in ALS subjects compared to HCs for several muscles of the thigh and mainly for leg muscles. By contrast, FF was elevated in few muscles, and mainly at the level of the thigh. The applied mixed effects model showed that FF increased significantly in the leg muscles over time (mainly in the triceps surae) and that wT2 decreased significantly in line with worsening in the leg subscore of ALSFRS-R, mainly at the leg level and in the anterior and medial compartment of the thigh.

**Conclusions:** Quantitative MRI represents a non-invasive tool that is able to outline the trajectory of pathogenic modifications at the muscle level in ALS. In particular, wT2 was found to be increased early in the clinical history of ALS and also tended to decrease over time, also showing a positive correlation with leg subscore of ALSFRS-R.

## Introduction

Amyotrophic lateral sclerosis (ALS) is a neurodegenerative disease that targets motor neurons, though other cells (including muscle cells) may be affected as well. Muscle involvement in ALS is traditionally considered to be secondary to neuronal damage, and this line of reasoning is recently being proven on qualitative and quantitative MRI (qMRI) sequences (1–7).

The precise and efficient evaluation and tracking of changes related to the evolution of the disease, beyond the traditional clinical measures (i.e., the revised ALS functional rating scale, ALSFRS-R), is a major difficulty in ALS. Quantitative muscle MRI has been proposed and successfully used to provide biomarkers for tracking disease evolution and, when available, assess response to treatment in neuromuscular disorders (8–11) and for ALS specifically (12). Longitudinal studies investigating the role of muscle MRI as a biomarker of ALS progression are few and heterogeneous in terms of evaluated MRI studies (1, 3, 4, 13).

As for T2 times, a study by Jenkins et al. found that T2 signal was increased in ALS subjects compared to controls, with muscle differences as low as 0.6% (for right trapezius) and as high as 71.4% for tibialis anterior (TA); T2 signal also increased over time in ALS subjects at 4-month follow-up for TA (3). In a more recent study, additionally, T2 also increased at 12-month follow-up for right TA, right quadriceps, bilateral hamstrings, and gastrocnemius/soleus (by a 14–29%) (4). They also found that increase in T2 signal after 1 year correlated with progressive weakness and with the loss of motor units at EMG (4).

In the present study, our aim was to assess changes in trophicity, fat replacement, and intramuscular edema of lower limbs in the early phases of ALS, and to explore the eventual correlation with clinical scores, with a longitudinal approach. Measurements of FF, cross-sectional area (CSA), and water T2 (wT2) were performed to evaluate the intramuscular changes at baseline and follow-up.

## Materials and Methods

### Subjects

Patients newly diagnosed with ALS using the El Escorial Criteria (14) were recruited into this longitudinal study from patients who attended the motor neuron disease clinic at our Institution. Exclusion criteria consisted of an inability to give informed consent, concomitant neuromuscular diseases, respiratory failure impairing ability to lie still in the scanner, and safety-related MRI contraindications. We scored clinical severity by using the subitems of the ALSFRS-R (15), indicating lower limb motor function (walking and climbing stairs). Each patient underwent a full clinical assessment, including the taking of medical history and an evaluation of clinical and neurologic conditions. Patients were clinically evaluated at each time point (baseline, t1 after 6 months, and t2 after 12 months). Eleven healthy controls (HCs) were enrolled for comparison.

All subjects gave written informed consent for participation in this study, which was approved by the local Ethical Committee.

### Imaging Protocol

At each clinical time point (t0, t1, and t2), subjects underwent a quantitative muscle MRI protocol (3T Skyra scanner, Siemens Healthcare, Erlangen, Germany) with an 18-channel phased-array coil applied on thighs and legs. The acquisition protocol included two quantitative sequences acquired in the axial plane both for the thigh and the leg: a 6-point Dixon gradient-echo (GRE) sequence (voxel size: 1 mm × 1 mm × 5 mm; slice thickness: 5 mm, TR: 35 ms, TE: 1.73 ms) and a multi-echo turbo spin echo (TSE) T2 sequence (voxel size: 1.2 mm × 1.2 mm × 10 mm; slice thickness: 10 mm, TR: 4,100 ms, TE: 10.90 ms, 17 echoes). Vitamin E capsules were placed upon the skin of the patient as positioning markers to allow cross-sectional intra-cohort reproducibility and longitudinal intra-subject reproducibility. The total duration of muscle MRI protocol was approximately 45 min. Some patients were lost to longitudinal follow-ups due to physical inability (major clinical impairment) to perform the muscle MRI protocol and logistic/personal issues linked to the COVID-19 pandemic. In the end, a total of 15 ALS subjects underwent muscle MRI at t0, nine at t1, and seven at t2. HCs only underwent a single muscle MRI examination.

### MRI Data Post-Processing

**Figure 1:**
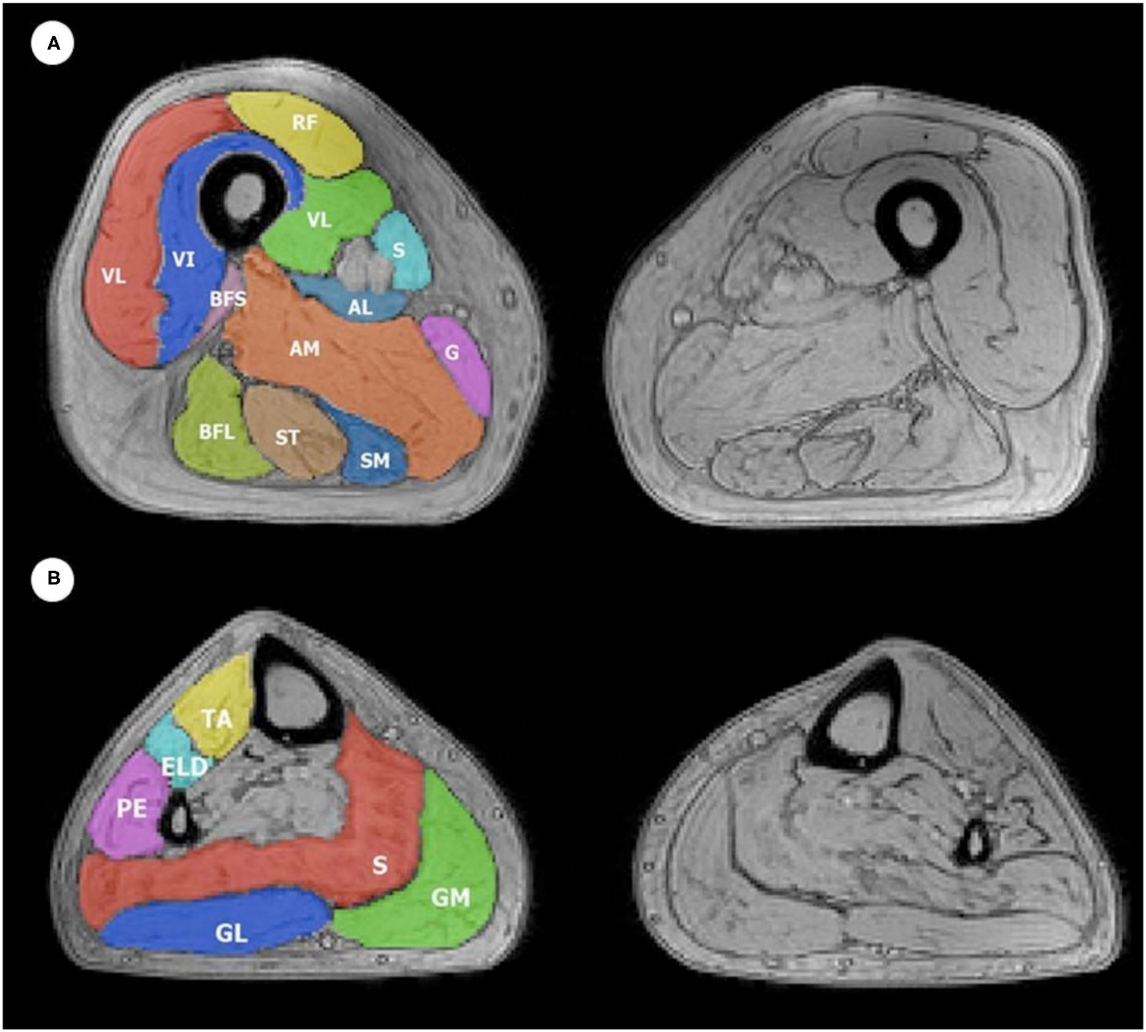
Example of ROIs obtained during manual **(A)** thigh and **(B)** leg muscle segmentation superimposed on a T2w GRE image. The color code indicates all 12 thigh ROIs and six leg ROIs that were considered, which were drawn on both the left and right thigh, with the corresponding muscle indicated in the figure with its respective initials, as follows: **(A)** Thigh muscles: VL, vastus lateralis; VM, vastus medialis; VI, vastus intermedius; RF, rectus femoris; Sa, sartorius; G, gracilis; AM, adductor magnus; SM, semimembranosus; ST, semitendinosus; BFL, long head of biceps femoris; BFS, short head of biceps femoris; AL, adductor longus. The anterior compartment includes VL, VM, VI, and RF; medial compartment includes Sa, G, AM, and AL; the posterior compartment includes SM, ST, BFL, and BFS. **(B)** Leg muscles: TA, tibialis anterior; ELD, extensor digitorum longus; PE, peroneus; S, soleus; GM, medial head of the gastrocnemius; GL, lateral head of the gastrocnemius. The anterior compartment includes TA, ELD, and PE; the posterior compartment includes S, GM, and GL.

The segmentation of thigh and leg muscles was performed through the region of interest (ROI) drawing by a single expert operator (with 5 years of experience in muscle segmentation), blinded to clinical data, using the open-source software ITK-SNAP (16). A total of 12 thigh and six calf muscle ROIs for each side (left and right) were evaluated (Figure 1).

Muscle ROIs were drawn without including non-muscle tissues, such as fat tissue, fascia, or blood vessels. Muscle perimeter was outlined approximately 1 mm inside the borders of the perimuscular fascia. ROIs were initially drawn on a single median slice of the first echo image of the multi-echo T2 sequence. The ROIs were then registered on the seven most median slices of the GRE images using linear and non-linear transformations. These were subsequently manually revised and corrected by the same operator to avoid registration errors.

The average fat fraction (FF) was calculated for every muscle from the 6-point Dixon GRE images, applying the publicly available post-processing algorithm FattyRiot (17). Accordingly, the global T2 value was calculated from the multi-echo T2 images. wT2 was then calculated from the multi-echo spin-echo T2 acquisition using a two-compartment extended-phase-graph fitting (18, 19). CSA was also calculated for each ROI and used for analysis.

### Statistical Analysis

Statistical analyses were performed using R 4.0.2 (20). Descriptive statistics present continuous variables as mean and SD, or median and range where appropriate, whereas categorical variables are shown as row counts and percentages. The level of significance was set at *p* < 0.05. The comparison between patients and HCs at baseline was performed using the Wilcoxon test for independent samples.

In order to include all the available data in the analysis of time influence on variables involved, linear mixed models were implemented where time was included as a numeric explanatory variable. The evaluation of correlation with the clinical scale was performed through linear mixed models where time and clinical scale were included as numeric explanatory variables.

Furthermore, a principal component analysis (PCA) was performed to aggregate information of muscle groups (anterior, medial, and posterior compartment of the thigh, anterior, and posterior compartment of the leg) for FF, wT2, and CSA. For subdivision into compartments of the thigh and for the leg, refer to the legend of Figure 1. The Pearson correlation analysis was performed between the first principal component of each group and the clinical scale. The analyses were performed on timepoints 0 and 1 and the differences.

## Results

### Demographics and Clinical Findings

About 15 patients with ALS (M/F = 8/7; average age 62.2 years old, range 29–79 years) newly diagnosed with possible (*n* = 2), probable (*n* = 12), or definite (*n* = 1) ALS were enrolled for this study. All patients presented sporadic ALS. Eleven patients presented spinal onset, whereas four of them had initial bulbar involvement. Mean diagnostic delay (time from symptoms onset to diagnosis) was 12.6 months (range, 3–30), and the mean ALSFRS-R score was 43.4 (range, 38–47). Mean ALSFRS-R score of subitems related to leg involvement were 6.6 (range, 2–8) at T0, 5.4 (range, 3–8) at T1, and 5.1 (range, 1–8) at T2. Clinical data for each patient are reported in Table 1. HCs (M/F = 4/7) had an average age of 52.8 years (range 42–72).

**Table 1:** Clinical and epidemiological data.

**Patient ID (sex, age at onset—years)**	**El Escorial Category**	**Site of onset**	**Type of involvement**	**ALSFRS-R score at diagnosis (leg subscore)**	**ALSFRS-R score at T1 (leg subscore)**	**ALSFRS-R score at T2 (leg subscore)**	**Predominant UMN/LMN**
#1 (F, 55)	Probable	Spinal	Distal left LL	44 (7)	–	36 (5)	LMN
#2 (F, 39)	Definite	Spinal	Proximal left and right LL	38 (6)	35 (4)	33 (4)	UMN
#3 (M, 29)	Probable	Spinal	Distal left UL	47 (8)	45 (7)	–	LMN
#4 (M, 63)	Probable	Spinal	Proximal and distal left LL	41 (4)	36 (3)	31 (1)	LMN
#5 (M, 75)	Possible	Spinal	Proximal left UL	46 (8)	–	–	LMN
#6 (M, 74)	Possible	Spinal	Proximal left and right UL	45 (7)	39 (3)	34 (2)	LMN
#7 (F, 66)	Probable	Bulbar	–	40 (8)	38 (8)	37 (8)	LMN
#8 (M, 56)	Probable	Spinal	Proximal-distal, left-right UL-LL	45 (2)	–	–	LMN
#9 (M, 79)	Probable	Spinal	Distal left LL	43 (5)	37 (4)	–	LMN
#10 (M, 73)	Probable	Bulbar	–	44 (8)	–	40 (8)	LMN
#11 (F, 63)	Probable	Bulbar	–	44 (8)	41 (7)	–	LMN
#12 (F, 76)	Probable	Bulbar	–	42 (8)	40 (8)	–	LMN
#13 (M, 69)	Probable	Spinal	Distal right LL	45 (7)	40 (5)	–	LMN
#14 (F, 64)	Probable	Spinal	Distal right UL	42 (6)	–	–	LMN
#15 (F, 52)	Probable	Spinal	Proximal right LL	45 (8)	–	39 (8)	UMN

*LL, lower limb; UL, upper limb; LMN, lower motor neuron; UMN, upper motor neuron*.

### qMRI Measures Differences Between ALS and Healthy Controls

At baseline, compared to HCs, wT2 was significantly increased in ALS subjects in several muscles of the thigh, including anterior, medial, and posterior compartment; at the level of the leg, wT2 was increased in ALS subjects for TA, triceps surae, extensor longus digitorum, and peroneal muscles (Table 2). CSA was decreased for ALS subjects only for semimembranosus muscle; by contrast, FF was increased in ALS subjects mainly at the level of the anterior and medial compartment of the thigh and in ELD and TA at the leg level (Table 2).

**Table 2:** Differences in w-T2, CSA and FF between ALS patients and healthy control at baseline.

**ALS vs. HCs**				
**Variables**		**Mean difference**	**Median difference**	***p*** **-value**
wT2	VL_left-right^*^	+2.809572	+3.063086	0.00371575
	VI_left-right^*^	+2.897702	+1.696587	0.00211737
	RF_left-right^*^	+2.289581	+1.427143	0.02141274
	AM_left-right^*^	+2.879314	+3.131731	0.00173943
	SM_left-right^*^	+3.784083	+4.275337	0.00256606
	ST_left-right	+2.339892	+3.364537	0.01192037
	ST_left	+2.483095	+3.026519	0.01020843
	BFL_left-right^*^	+3.796152	+2.774935	0.00740422
	BFS_left-right	+4.148411	+4.909326	0.01020843
	BFS_left	+5.796794	+4.527675	0.00309437
	So_left-right^*^	+7.020631	+5.605596	0.00000771
	MG_left-right^*^	+10.8707	+9.009979	0.00000081
	LG_left-right^*^	+11.80878	+13.24945	0.00000771
	TA_left-right^*^	+8.202594	+6.730464	0.00001116
	ELD_left-right^*^	+8.375868	+8.214133	0.00004280
	Pe_left-right^*^	+8.861914	+8.65537	0.00002243
CSA	SM_left-right	−374.509	−464.145	0.02814128
	SM_left-right	−202.811	−246.28	0.01371954
FF	RF_left-right	+0.033108	+0.037	0.00228725
	VM_left	+0.021782	+0.02615	0.04686635
	RF_left	+0.032167	+0.03425	0.01114513
	Sa_left	+0.041602	+0.04835	0.01858989
	AM_left	+0.037055	+0.0384	0.03211220
	VI_right	+0.018415	+0.01955	0.03376167
	BFS_right	+0.119552	+0.14275	0.01608458
	ELD_left-right	+0.036058	+0.0259	0.03806963
	TA_left	+0.031363	+0.02125	0.02814128
	ELD_left	+0.0409	+0.0258	0.04143832

*For mean and median values, positive values indicate ALS > HCs, negative values indicate ALS < HCs. Muscle abbreviated names as in Figure 1*.

* *Only results related to the mean value are reported when difference of right, left and mean features are significant*.

### Longitudinal Evolution of qMRI Measures and Correlation to Leg Subscore of ALSFRS-R

When evaluating the evolution of qMRI measures over time, we found that wT2 decreased significantly over time only in the gemellus medialis and peroneal muscles. By contrast, FF increased over time in gemellus medialis, soleus, and TA. No significant change of FF was found over time for thigh muscles (Table 3). No significant change was observed for CSA overall.

**Table 3:** Longitudinal qMRI evaluation of ALS subjects—mixed effects models results when time is the explanatory variable of the model.

**Variables**		**Coefficients**	**Std errors**	***P*** **-value**
wT2	MG_left	−0.17168	0.0765288	0.0404
	Pe_right	−0.38271	0.159210	0.0296
FF	So_left-right	+0.00294898	0.001113378	0.0182
	So_left	+0.00328409	0.00148746	0.0432
	So_right	+0.00259289	0.001047599	0.0257
	MG_left-right	+0.00318101	0.001027389	0.0074
	MG_right	+0.00366893	0.001304145	0.0131
	TA_left	+0.00137111	0.000594534	0.0358
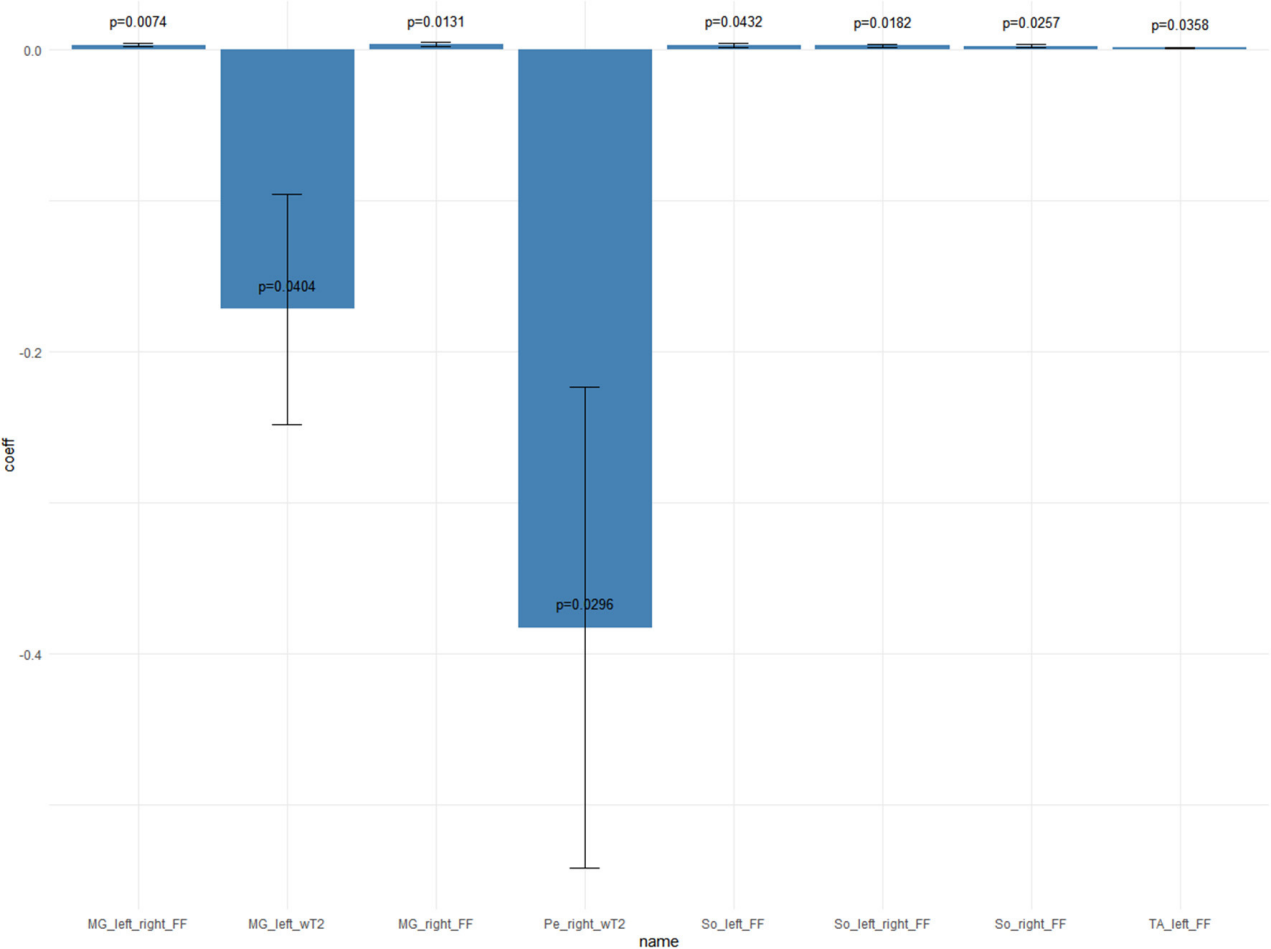				

*wT2 decreased over time for gemellus medialis and peroneal muscles; FF increased over time in gemellus medialis, soleus and tibialis anterior. No correlation was found for thigh muscles. Coefficients, standard errors and p-values are reported*.

*Bar plot represents the magnitude of coefficients in the linear mixed model. Error bars represent the standard error of coefficients and the significance of the coefficient is reported on the bar. The height of each bar represent the influence of the time on the single variable, weighted with respect to the error, in the model that consider time as the explanatory variable. For each muscle acronym please see Figure 1*.

In an attempt to correlate qMRI measures and ALSFRS-R scale scores, with time considered as adjustment for longitudinal data, we found that neither FF nor CSA showed any significant correlation. By contrast, wT2 showed a positive correlation with the clinical scale in several muscles of the thigh including the anterior, medial, and posterior compartment (i.e., the higher the muscle wT2, the higher the ALSFRS-R scale) (Table 4).

**Table 4:** Longitudinal correlation of leg subscore of the ALSFRS-R scale with qMRI measures—mixed effects models result.

**Variables**		**Coefficients**	**Std Error**	***p*** **-value**
wT2	VM_left	+0.241773	0.110780	0.0480
	RF_left-right	+0.243178	0.092209	0.0205
	RF_right	+0.240500	0.087082	0.0162
	Sa_left-right	+0.1569837	0.0544809	0.0129
	Sa_left	+0.1264864	0.0447453	0.0143
	Sa_right	+0.1300158	0.0507045	0.0236
	G_left-right	+0.254595	0.078055	0.0062
	G_left	+0.1422255	0.0639267	0.0444
	G_right	+0.321866	0.083583	0.0020
	AM_left	+0.325993	0.119467	0.0172
	ST_left-right	+0.326414	0.119586	0.0172
	ST_left	+0.321915	0.122695	0.0210
	ST_right	+0.248465	0.103604	0.0322
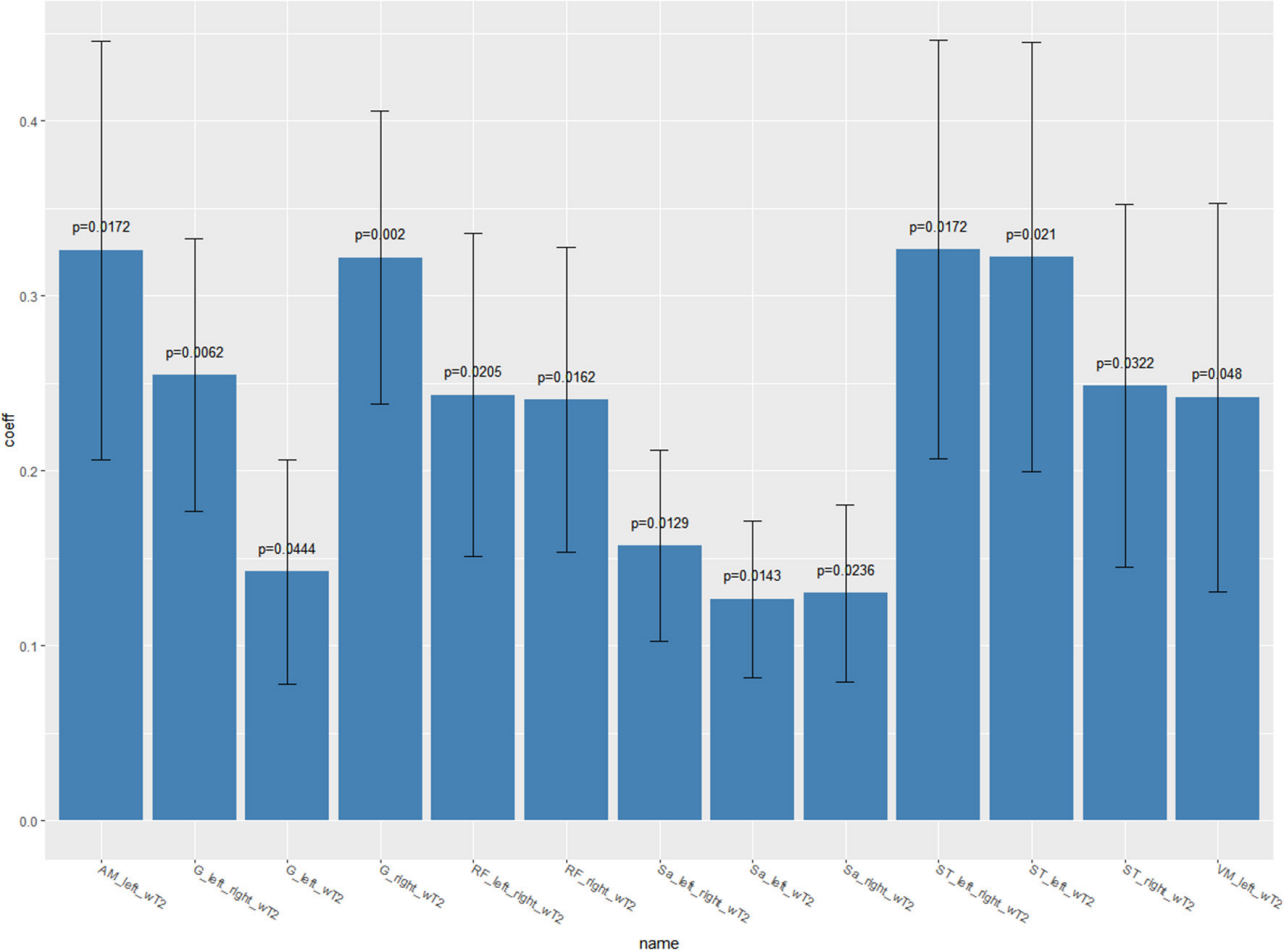				

*ALSFRS-R scale is the explanatory variable of the model, with time considered as adjustment for longitudinal data. Only water-T2 mainly showed a modest positive correlation with the clinical scale. Coefficients, standard errors and p-values are reported*.

*Bar plot represents the magnitude of coefficients in the linear mixed model. Error bars represent the standard error of coefficients and the significance of the coefficient is reported on the bar. The height of each bar represent the influence of leg subscore of the ALSFRS-R scale on the single variable, weighted with respect to the error, in the model that consider the scale and the time as explanatory variables. For each muscle acronym please see Figure 1*.

The PCA was performed to aggregate information of muscle compartments (see Figure 1) for either FF, wT2, or CSA and did not show any significant result (*p* > 0.05).

## Discussion

In the present study, we performed quantitative muscle MRI of lower limbs in a monocentric cohort of early-onset ALS subjects, assessing fat infiltration, trophicity, and intramuscular edema.

In the cross-sectional analysis, we showed that the wT2 signal was increased in ALS subjects for several muscles of both thigh and leg as early as the diagnostic phase of the disease. FF, and especially CSA, did not show any significant changes. In the longitudinal follow-up, we found that the wT2 signal decreased and FF increased in some leg muscles; no significant changes were found in thigh muscles. Furthermore, no changes were found for CSA over time both for thigh and leg muscles.

In our cohort, the median time between symptoms onset and diagnosis was 12.6 months, in accordance with the literature data. Thus, we assumed that enrolled ALS subjects were in the initial phases of muscle involvement when the denervation process was evident, trophicity was still relatively preserved, and fatty replacement was absent. Our finding of increased wT2 confirmed the ability of qMRI to depict consequences of denervation in muscles, as already showed by Klickovic et al. (13). This finding is also consistent with the pathological process of muscle in patients with ALS, both in the early stages and during the trajectory of the disease (13).

The changes in wT2 in FF found in this study can be considered as rather symmetric and extensive throughout the lower limbs. Although we did not include other muscles of the body (or apply whole-body MRI) in our qMRI study, we agree with Jenkins et al. that lower limbs can be regarded as highly representative of the whole picture of muscle involvement in ALS (4). The relative degree of symmetry found in our study was also somehow inconsistent with the asymmetry of the clinical phenotype of the disease. We presume that MRI might be able to mirror the pathogenic pattern of ALS (i.e., diffuse involvement of all muscles, even at different time points), as it quantifies subtle changes in the fat and free water component. By contrast, the clinical assessment is likely influenced by several factors (such as the number of motor neurons involved, which may be different for each muscle).

When we attempted to correlate qMRI measurements with clinical measures of disease severity, we found a positive correlation between wT2 and ALSFRS-R leg subitems of walking and climbing stairs (i.e., leg subscore). At first sight, this finding may be confounding, as it seems to confirm that functional impairment of patients with ALS is mild when the denervation is predominant. However, we expected this finding, and we explain it with the fact that clinical impairment is not prominent in the first phases of the disease (where active denervation and wT2 increase occurs), whereas when the ability to walk and climb stairs decreases (i.e., ALSFRS-R leg subscore decreases) wT2 is reduced. By contrast, FF increases over time only in leg muscles and not in thigh muscles. The ALS subjects included in this study presented with predominant involvement of distal leg muscles (e.g., Patrikios subtype, refer to Table 1); in parallel, we can assume that FF increased earlier in the leg muscles than in the thigh muscles. Furthermore, our imaging follow-up can be considered quite short (up to 12 months) and presumably FF was not yet altered in the thigh muscles due to the slow progression of this subtype of ALS.

As for existing literature on fat replacement and edema-like changes in the muscle of ALS subjects, the former approaches involved conventional wT1 and Short tau inversion recovery (STIR) sequences. Even recently, Klickovic et al. proposed a mixed qualitative and quantitative MRI analysis of bulbar and lower limb muscles in order to differentiate ALS and spinal bulbar muscular atrophy (SBMA), applying FF calculation, in addition to a semiquantitative evaluation of T1-weighted and STIR sequences (13). With specific regard to the second point, the authors demonstrated an extensive involvement of the lower limb muscles in ALS, reflecting edema-like changes, which are signs of denervation processes, observed as areas of STIR hyperintensity (13, 21, 22).

Already in 1998, Bryan et al. approached T1 and T2 relaxometry in a cohort of 11 ALS subjects, demonstrating a robust negative correlation between T2 relaxation time and maximal voluntary isometric contraction (MVIC) and compound muscle action potential amplitude (CMAPa) (1).

A recent study by Jenkins et al. showed that the T2 signal in the TA increased over time in ALS subjects (3). The same group, using whole-body qMRI, showed in 2020 that leg muscles T2 changes were the most effective biomarkers in their cohort of ALS subjects, independent of the clinical onset location, and that slow progressor (i.e., patients with a slow disease) had detectable changes over time when assessing T2 signal (4).

The technical approach that was employed, nonetheless, was limited by the fact that no FF measurements, or calculation of real edematous changes in the muscle, were performed (i.e., wT2) separately from the fat signal (9, 23). The T2 relaxation time is in fact influenced by both intramuscular edema and fat replacement, making it difficult to distinguish the most prominent ongoing pathological manifestation in the muscle structure at the moment of MRI assessment (i.e., when edematous changes or rather a fat infiltration prevails over the other).

In our opinion, the technical approach used by these two experiences, which included T2 relaxation time (i.e., a global T2 time), did not fully allow the understanding of edematous or edema-like changes in the muscle, as fat components presumably had an impact on the results. On the contrary, with the robust technical approach we used in this study, which allowed us to depurate the fat component from the T2 signal of the muscle, we showed that wT2 was increased in ALS subjects, with a decrease over time and that it also had a positive correlation with clinical status as expressed by the ALSFRS-R.

We also recognize, however, that our study protocol can be physically challenging to endure for ALS subjects, and for this reason, we decided to concentrate only on lower limb muscles, due to the fact that simultaneous evaluation of FF and wT2 significantly prolong scanning time for each site.

### Study Limitations

The current study is limited in some regards. First, our data is limited by the small number of subjects enrolled for the MRI examination; such a number has been influenced by the rarity of the disease and by our intent to perform a comprehensive and robust (though quite long) quantitative muscle MRI protocol on the same scanner. The SARS-COVID-19 epidemic had also an impact on the enrollment of research patients during 2020.

Second, due to the difficulty of acquiring MRI scans in a cohort of ALS subjects, despite enrolling patients at their clinical onset, we lost many of them at follow-up appointments. This happened due to clinical reasons, the impossibility to maintain the position for the adequate time for scanning, and also for the death of the subject.

Third, the lack of a recognized connection between the qMRI data and the currently used clinical scales (e.g., the leg subscore of the ALSFRS-R score used in this study) may have at least partially limited the possibility to extend the validity of the presented data. This issue, however, can be considered as a minor element, due to the recently recognized importance and significance of quantitative muscle MRI features (as FF, wT2, etc.) in neuromuscular diseases and other pathologies. We are also planning to expand the current data with a correlation between qMRI and electromyography (EMG) to gain a deeper insight into the pathology.

## Conclusions

In conclusion, despite the small cohort examined, our study demonstrated that wT2 was increased in the early phases of ALS. Furthermore, wT2 correlated positively with the leg subscore of the ALSFRS-R and decreased over time. Such results encourage us to continue applying qMRI in the assessment of muscle damage in ALS. Future studies with larger cohorts are needed to confirm and expand these preliminary data.

## Data Availability Statement

The original contributions presented in the study are publicly available. Raw MRI data are available in the Zenodo repository: https://zenodo.org/record/5575243#.

## Ethics Statement

The studies involving human participants were reviewed and approved by Comitato Etico Pavia. The patients/participants provided their written informed consent to participate in this study.

## Author Contributions

LD, MP, and AP contributed to study design, data analysis, writing of the manuscript, and critical revision of the manuscript. XD, FSa, and NB contributed to data acquisition and analysis, and critical revision of the manuscript. FSo, SM, and LF contributed to data analysis. EB and SF contributed to statistical analysis. All authors gave important contributions to the final form of the manuscript.

## Funding

This work was supported by the Italian Ministry of Health (RC 2017–2021).

## Conflict of Interest

The authors declare that the research was conducted in the absence of any commercial or financial relationships that could be construed as a potential conflict of interest.

## Publisher's Note

All claims expressed in this article are solely those of the authors and do not necessarily represent those of their affiliated organizations, or those of the publisher, the editors and the reviewers. Any product that may be evaluated in this article, or claim that may be made by its manufacturer, is not guaranteed or endorsed by the publisher.
